# Clinical therapeutic effects of combined diacerein and glucosamine in the treatment of osteoarthritis: A protocol for systematic review and meta-analysis

**DOI:** 10.1097/MD.0000000000027583

**Published:** 2021-11-24

**Authors:** Fei Wang, Wei-Xing Shi, Jie Chen, Kang He, Wei Fang

**Affiliations:** No.1 Orthopedics Department, Ezhou Central Hospital, Ezhou City, Hubei Province, China.

**Keywords:** diacerein, efficacy, glucosamine, meta, osteoarthritis

## Abstract

**Background:**

Osteoarthritis (OA) has been identified as a common musculoskeletal condition. As a chronic condition, OA adversely impact the hip and knee joints. Surgical treatment for hip and knee osteoarthritis is associated with high financial and long recovery processes. Therefore, patients are continually searching for alternative methods of treatment. Diacerein is regarded as symptom-modifying, slow-acting drug that could most likely change the disease structure of OA. The present systematic review protocol explains methods utilized to evaluate the clinical therapeutic effects of combining diacerein and glucosamine to treat OA.

**Methods:**

The authors will conduct a search for randomized controlled trials comparing diacerein plus glucosamine with diacerein alone, glucosamine alone, or another treatment in patients with OA. The search will be done in the following online-based databases: EMBASE, MEDLINE, Cochrane Library, Web of Science, China National Knowledge Infrastructure, and WanFang Database. All related RCTs included from inception to September 29, 2021 are included. Two authors will independently conduct data abstraction and quality assessment, and the comparative analysis will compare the results. The present meta-analysis will be performed with the RevMan software (version 5.3), where the results will be expressed as relative risk, mean differences, or standardized mean differences with 95% confidence intervals.

**Results:**

This study will be conducted to evaluate the clinical therapeutic effects of combined diacerein and glucosamine in the treatment of OA.

**Conclusion:**

The summary presented in the study will ascertain whether diacerein plus glucosamine intervention is an efficient and feasible method of treatment for OA patients.

**Trial registration number:**

10.17605/OSF.IO/VHPZC

## 1 Introduction

Osteoarthritis (OA) is prevalent among the elderly population. It is a progressive and chronic musculoskeletal condition.^[1,2]^ Initially, OA patients experience deterioration in the cartilage resulting in inflammation and destruction of the joints surrounding tissues, including the cartilage, synovium, and bone.^[3–5]^ It is not possible to restore the health of the damaged cartilage, and therefore, it is essential to properly manage OA. Generally, OA treatment is primarily directed at alleviating pain and elevating functionality. Current OA therapies including analgesics, non-steroidal anti-inflammatory drugs (NSAIDs), and surgical procedures. Indeed, these methods are effective at treating the symptoms of OA.^[6]^ However, they are palliative and cannot the progression of the disease.^[7]^ Reportedly, several agents and compounds have shown potential in reducing the severity of the condition, including its symptoms.

It has been established that diacerein can improve disease modifying anti-catabolic, anti-inflammatory, and pro-anabolic events on the cartilage and synovial fluid of joints through inhibitory action on interleukin-1β.^[8–11]^ Moreover, diacerein is related to reduced off-target effects in relation to the severe side effects of NSAIDs. Presently, the clinical management of OA entail a combination of treatment options to minimize the pain and raise functional activities. The European Society for Clinical and Economic Aspects of Osteoporosis and Osteoarthritis advocated a higher benefit to risk ratio of using diacerein to treat the symptoms of OA and placed it as a front-line agent for those who have issues with NSAIDs, with lasting (several months) carry-over effects following the discontinuation of therapy.^[12]^

It has been reported that combining diacerein with another treatment exhibited higher effectiveness when treating OA patients.^[11,13]^ Thus, it is crucial to perform a meta-analysis on the clinical therapeutic effects of combining diacerein and glucosamine in the treatment of OA.

## 2 Methods

The present protocol is registered on the Open Science Framework (OSF, https://osf.io/), the registration number is 10.17605/OSF.IO/VHPZC. The development of the protocol will follow the guidelines of Preferred Reporting Items for Systematic Reviews and Meta-Analyses protocols (PRISMA-P).^[14]^

### 2.1 Ethics and dissemination

Since the systematic evaluation does not collect patient information and uses the data from published research papers, the proposed meta-analysis will not need an ethics approval and informed consent of patients.

### 2.2 Eligible criteria for study selection

#### 2.2.1 Types of studies

Only randomized controlled trials (RCTs) of diacerein plus glucosamine in treating OA patients are considered.

#### 2.2.2 Types of participants

All participants with OA are included, there are no restrictions for age, sex, country, and educational background.

#### 2.2.3 Types of interventions

This study includes diacerein plus glucosamine to provide a detailed description of its clinical therapeutic effects on OA patients. Studies that are interfered with diacerein alone, glucosamine alone, no treatment, placebo, or other traditional drugs are considered as control groups.

#### 2.2.4 Types of outcome measures

The primary outcomes include physical function, pain, Lequesne index, radiographic outcomes (radiologic differences in the width of the joint space or narrowing in millimeters, and alternative radiographic standards), collective number of negative effects/events (AEs), and serious AEs. The minor outcomes include quality of life, requirement for joint surgery or arthroscopy, requirement for concomitant medications for number of fatalities, pain relief, and specific AEs.

### 2.3 Search methods for the identification of studies

#### 2.3.1 Electronic searches

The authors will conduct a search for all RCTs that compared diacerein plus glucosamine with diacerein alone, glucosamine alone, or another treatment in patients with OA. The search will be done in the following online-based databases: EMBASE, MEDLINE, Cochrane Library, Web of Science, China National Knowledge Infrastructure, and WanFang Database. All related RCTs included from inception to September 29, 2021 are included. The authors will use the following key terms for searching: osteoarthritis, OA, diacerein, glucosamine, pain, function, score, grade, motion.

#### 2.3.2 Searching other resources

The authors will search the reference lists of the eligible studies to find additional trials. Since there are several international nutraceutical companies in the chondroitin market, it was deemed that contacting each one to gain unpublished data would not be feasible. This study will not include any search conference proceedings or specific journals, as defined.

### 2.4 Data collection and analysis

#### 2.4.1 Selection of studies

Pre-determined criteria will be adopted to identify potentially eligible trials. A pair of authors will autonomously evaluate the methods sections in all identified trials based on the pre-determined assessment criteria. All disagreements will first be resolved by consensus. For unresolved disagreements, a third independent author's opinion would be considered.

#### 2.4.2 Data extraction and management

We will use an electronic form to extract crucial content, and then it will be filled by 2 authors autonomously. The content include first and corresponding author, inception time, trial designate, features of patients, intercessions, duration, outcomes, adverse impacts, and other specific information. Disagreements shall be solved via group discussion or by consulting with seniors. However, if the authors do not reach a consensus, the respective authors of the studies will be contacted to obtain additional details and further verification.

#### 2.4.3 Assessment of risk of bias in included studies

Two independent authors shall assess the bias risk associated with each trial included with the Cochrane Collaboration assessment recommendations. The primary criteria applied in measuring the risk of bias include blinding presence, allocation concealment, random sequence generation, partial outcome data, and selective outcome reporting.^[15]^ The bias risk of each study was assessed under each criterion using the following standard: low bias risk, high bias risk, or unclear bias risk. Disagreements shall be resolved through a group discussion or by consulting seniors.

#### 2.4.4 Measures of treatment effect

Continuous data will be represented using the mean differences, or standardized mean differences with 95% confidence intervals when evaluating the treatment effect, under which a similar method or measure scale are adopted. For dichotomous data, the relative risk with 95% confidence interval will be used to estimate the treatment effect.

#### 2.4.5 Assessment of heterogeneity

The authors will qualify inconsistencies in pooled estimates by adopting the *I*^*2*^ statistic. It will illustrate the variability percentage in effect estimates that originate from heterogeneity instead of the sampling error.^[15]^

#### 2.4.6 Assessment of reporting biases

A funnel plot will be employed to determine the publication bias if there are >10 eligible studies for inclusion.^[16]^

#### 2.4.7 Sensitivity analysis

In order to ascertain the robustness of the conclusions, we will conduct a sensitivity analysis according to the following criteria: sample size, qualities of heterogeneity, and statistical model (random-effects or fixed-effects model).

## 3 Discussion

To the best knowledge of the authors, this meta-analysis is the first to assess the clinical therapeutic effects of using combined diacerein and glucosamine to treat OA. Indeed, similar articles discuss the issue. However, they did not include non-RCT studies, they also did not include papers written in different languages. Therefore, the results of the present study will contribute to the literature and fill the gap in research. Besides, it will provide reference for clinical practice.

## Author contributions

**Conceptualization:** Fei Wang, Wei-Xing Shi, Jie Chen, Kang He, Wei Fang.

**Data curation:** Fei Wang, Wei-Xing Shi, Jie Chen.

**Formal analysis:** Fei Wang, Wei-Xing Shi, Wei Fang.

**Funding acquisition:** Jie Chen, Kang He.

**Investigation:** Fei Wang, Wei-Xing Shi, Jie Chen, Kang He.

**Methodology:** Kang He.

**Project administration:** Fei Wang, Wei Fang.

**Resources:** Wei-Xing Shi, Jie Chen, Kang He.

**Software:** Fei Wang.

**Validation:** Fei Wang, Wei-Xing Shi, Jie Chen, Kang He, Wei Fang.

**Visualization:** Wei-Xing Shi, Kang He.

**Writing – original draft:** Fei Wang.

**Writing – review & editing:** Kang He.
